# Low pH-Induced Changes of Antioxidant Enzyme and ATPase Activities in the Roots of Rice (*Oryza sativa* L.) Seedlings

**DOI:** 10.1371/journal.pone.0116971

**Published:** 2015-02-26

**Authors:** Yi-Kai Zhang, De-Feng Zhu, Yu-Ping Zhang, Hui-Zhe Chen, Jing Xiang, Xian-Qing Lin

**Affiliations:** State Key Laboratory of Rice Biology, China National Rice Research Institute, Hangzhou, Zhejiang, 310006, P. R. China; Zhejiang University, CHINA

## Abstract

Soil acidification is the main problem in the current rice production. Here, the effects of low pH on the root growth, reactive oxygen species metabolism, plasma membrane functions, and the transcript levels of the related genes were investigated in rice seedlings (*Oryza sativa* L.) in a hydroponic system at pH 3.5, 4.5, and 5.5. There were two hybrid rice cultivars in this trial, including Yongyou 12 (YY12, a japonica hybrid) and Zhongzheyou 1 (ZZY1, an indica hybrid). Higher H^+^ activity markedly decreased root length, the proportion of fine roots, and dry matter production, but induced a significant accumulation of hydrogen peroxide (H_2_O_2_), and led to serious lipid peroxidation in the roots of the two varieties. The transcript levels of copper/zinc superoxide dismutase 1 (Cu/Zn SOD1), copper/zinc superoxide dismutase 2 (Cu/Zn SOD2), catalase A (CATA) and catalase B (CATB) genes in YY12 and ZZY1 roots were significantly down-regulated after low pH exposure for two weeks. Meanwhile, a significant decrease was observed in the expression of the P-type Ca^2+^-ATPases in roots at pH 3.5. The activities of antioxidant enzymes (SOD, CAT) and plasma membrane (PM) Ca^2+^-ATPase in the two varieties were dramatically inhibited by strong rhizosphere acidification. However, the expression levels of ascorbate peroxidase 1 (APX1) and PM H^+^-ATPase isoform 7 were up-regulated under H^+^ stress compared with the control. Significantly higher activities of APX and PM H^+^-ATPase could contribute to the adaptation of rice roots to low pH.

## Introduction

Soil acidification is one of the most serious environmental problems in intensive agricultural systems, mainly because of the excessive use of acidic and physiologically acidic nitrogen fertilizers and the acid rain caused by environmental pollution [1–3]. The pH value of most acidic soil significantly declined from the 1980s to the 2000s in the South China, and the pH is under 4.0 in some highly acidic soils [3]. Along with decreased crop productivity caused by low pH levels, the common causes of reduced yields include aluminum, manganese and hydrogen (H^+^) ions toxicities, and deficiencies in nutrients such as phosphorus, molybdenum, calcium, and magnesium [4]. Among these constraints, proton toxicity (low-pH stress) is considered to be one of the major stresses limiting plant growth in acid soils [5].

Low pH levels directly inhibited plant growth via high H^+^ activity [6, 7]. A high concentration of H^+^ triggers typical oxidative stress on plants by inducing the accumulation of excess reactive oxygen species (ROS), such as superoxide radicals (O_2_^−^•) and hydrogen peroxide (H_2_O_2_) in plant tissues [8, 9]. To counteract oxidative damage, plants have evolved complex antioxidant systems including antioxidant enzymes such as superoxide dismutase (SOD), catalase (CAT), peroxidases (POD), ascorbate peroxidase (APX), glutathione reductase (GR), dehydroascorbate reductase (DR), and antioxidants such as a-tocopherol, ascorbate, reduced glutathione [10, 11]. Studies have indicated that higher activity levels of antioxidant enzymes may contribute to better H^+^ tolerance by increasing the protective capacity against oxidative damage [12, 13].

The plasma membrane (PM) is an important barrier for plants to transport ions into root cells [14]. Plant PMs contain an H^+^-ATPase that plays an important role in the modulation of many environmental factors, including toxins, light, injury, mineral nutrients and other biotic and abiotic constrains [15–18]. The enzyme acts as a primary transporter by pumping protons out of the cell, and generates an H^+^ electrochemical gradient, thereby providing the driving force for the active influx and efflux of ions and metabolites across the plasma membrane [19]. Additionally, H^+^-ATPase contributes to keeping the cytoplasmic pH steady [16, 20]. Under stress conditions, the expression of different isoforms of PM H^+^-ATPase may be responsible for the pumping of H^+^ against the higher H^+^ electrochemical gradient at the low pH [18, 21].

Rice (*Oryza sativa* L.) is one of the main grain crops and is the staple food of over half the world’s population. Soil acidification is becoming the main barrier to rice production, and thus research on the regulation of rice root growth at low pH has great theoretical and practical value. In the present study, we analyzed the changes in root length and biomass, H_2_O_2_ content, antioxidative enzyme activities and lipid peroxidation at different pH levels. Additionally, to better understand the adaptation to a low pH medium, plasma membrane was isolated, and the roles of PM H^+^-ATPase, Ca^2+^-ATPase in rice (*Oryza sativa* L.) were also studied. The transcriptional levels of genes encoding PM H^+^-ATPase, Ca^2+^-ATPase and anti-oxidative enzymes were investigated by real-time quantitative PCR. An attempt was made to reveal the physiological mechanisms involved in the acclimation of monocotyledonous plant cells to low pH levels.

## Materials and Methods

### Plant culture and experimental design

All experiments were conducted at the experimental base of the China National Rice Research Institute in Fuyang City, China (30°03′N 119°57′E). Two hybrid rice (*Oryza sativa* L.) cultivars were used, Yongyou 12 (YY12, a japonica hybrid) and Zhongzheyou 1 (ZZY1, an indica hybrid). Rice seeds were obtained from a commercial company (Zhejiang Wuwangnong Seeds Co., LTD, Hangzhou, China). The seeds were surface-sterilized with 30% (v/v) H_2_O_2_ for 20 min and soaked for 12 h in distilled water. They were then germinated on moist filter paper in the dark for 2 days at 32°C. The germinated seeds were cultured in wrapped filter paper in distilled water until three leaves were visible. Uniform seedlings were selected and then transplanted into 5-L black plastic pots with five seedlings in each pot. The seedlings were cultured using Yoshida rice nutrient solution [22]. The nutrient solution was replaced every 3 days.

Treatments were given after the plants were pre-cultured for 2 weeks. The seedlings were cultured in the solution adjusted to pH 5.5 (the control), 4.5, and 3.5 by the addition of 0.1 M NaOH to raise, or 0.05 M H_2_SO_4_ to lower the pH. The pH was modified twice every day. Three biological replicates were performed for each treatment. The pots were arranged randomly in the glasshouse and re-positioned randomly every week. The experiment was carried out under natural conditions with an air temperature of 22–30°C during the day and 15–20°C during the night. All plants were sampled after 2 weeks of treatment. Samples for enzyme assays and RNA extraction were frozen immediately in liquid nitrogen, and stored at −80°C.

### Root sampling and analysis

Roots were carefully washed and then scanned with a scanner (Epson V700, China). During scanning, the roots were placed in a glass dish containing water to untangle them and minimize root overlap. Large root systems were divided into several root subsamples for adequate scanning. Root length and root surface area were quantified from digital images using WinRHIZO PRO 2013 (Regent Instruments, Quebec, Canada). Scanned roots were dried and weighed.

### H_2_O_2_ and malondialdehyde (MDA) contents assays

The H_2_O_2_ concentration was determined according to Patterson et al. [23]. This assay is based on the absorbance change of an H_2_O_2_-titanium complex at 415 nm, which is formed by the reaction of tissue-H_2_O_2_ with titanium tetrachloride. The level of lipid peroxidation in fresh leaves and roots was expressed as the MDA concentration and determined from 2-thiobarbituric acid (TBA) reactive metabolites [24].

### Antioxidant enzyme extraction and assay

Antioxidant enzyme activities were determined in 0.3 g of roots homogenized in 3 mL of an extraction solution containing 50 mM Na_2_HPO_4_-NaH_2_PO_4_ buffer (pH 7.8), 0.2 mM EDTA and 2% insoluble polyvinylpyrrolidone in a chilled pestle and mortar. The homogenate was centrifuged at 12,000 × g for 20 min and the supernatant was used to determine enzyme activity. The entire extraction procedure was carried out at 4°C. All spectrophotometric analysis was conducted on a Beckman DU-800 spectrophotometer. Total SOD activity was assayed by the photochemical method described by Rao and Sresty [25]. One unit of enzyme activity was defined as the amount of enzyme required for 50% inhibition of the rate of nitro blue tetrazolium reduction measured at 560 nm. CAT activity was measured according to the method of Cakmak and Marschner [26] by measuring the decrease in absorbance at 240 nm to determine the disappearance of H_2_O_2_. The reaction mixture contained 25 mM phosphate buffer (pH 7.0), 10 mM H_2_O_2_ and 0.1 ml enzyme extract. APX activity was measured according to Nakano and Asada [27] by monitoring the rate of ascorbate oxidation at 290 nm. The assay mixture contained 0.25 mM AsA, 1.0 mM H_2_O_2_, 0.1 mM EDTA, and 0.1 ml enzyme extract in 25 mM phosphate buffer (pH 7.0).

### Plasma membrane isolation and ATPase activity assay

The PM was isolated from rice roots according to Kasamo [28]. Roots were ground in ice-cold homogenization buffer with a mortar and pestle. The homogenization buffer contained 25 mM HEPES-Tris (pH 7.2), 250 mM mannitol, 5 mM EDTA, 5 mM EGTA, 1 mM DTT and 1.5% (w/v) PVP. The isolation procedures were carried out at 4°C. The homogenate was filtered through four layers of cheesecloth and centrifuged at 560 × g for 12 min, and the supernatant was centrifuged at 10,000 × g for 15 min. The resulting supernatant was then centrifuged at 60,000 × g (Optima L-80 XP Ultracentrifuge; Beckman Coulter, Brea, CA, USA) for 30 min to yield a crude membrane fraction. The pellet was re-suspended with 1mL of a gradient buffer containing: 20 mM HEPES-Tris (pH 7.5), 5 mM EDTA, and 0.5 mM EGTA. The supernatant was layered on top of a step gradient consisting of 1mL of 45% and 33% (w/w) sucrose, and then centrifuged for 2 h at 70,000 × g. The PM-enriched fraction was collected at the 33%/45% sucrose interface. Each fraction was centrifuged for 1 h at 100,000 × g. The resulting pellet was re-suspended in a medium containing 20 mM HEPES-Tris (pH 7.5), 3 mM MgCl_2_, 0.5 mM EGTA, and 300 mM sucrose, and then quickly frozen in liquid nitrogen and stored at −70°C until used for enzyme assays. The protein was quantified according to the method of Bradford [29].

PM H^+^-ATPase activity was assayed as described by Briskin et al. [30]. The assay medium used for the present study contained 36 mM Tris-Mes (pH 6.5), 30 mM ATP-Na_2_, 3 mM MgSO_4_, 1 mM NaN_3_, 50 mM KNO_3_, 1 mM Na_2_MoO_4_, and 0.02% (v/v) Triton X-100, in the presence or absence of 2.5 mM Na_3_VO_4_. The reaction was started by adding 50 μL PM vesicles. After 30 min incubation at 37°C, the reaction was quenched by the addition of 55% (w/v) TCA. The H^+^-ATPase activity was determined by measuring the release of P_i_ [31]. PM Ca^2+^-ATPase activity was measured on the basis of the methods of He et al. [32]. Activity was expressed in μmol P_i_ mg^-1^ protein h^-1^.

### Total RNA extraction and real-time quantitative PCR (RT-qPCR)

Total RNA was isolated from frozen plant samples of all treatments using Trizol reagent (Takara, Tokyo, Japan). Purified RNA was quantified spectrophotometrically (Nanodrop 2000, Thermo Scientific, Wilmington, DE, USA). cDNA synthesis was performed with 2 μg of total RNA, oligo-dT(18), and superscript II reverse transcriptase (Promega, Madison, WI, USA) according to the manufacturer’s protocol in a total volume of 25 μL.

RT-qPCR was performed using a 7300 Real Time PCR System (Applied Biosystems, Foster City, CA, USA) and a SYBR Green PCR Master Mix Kit (Toyobo Co., Osaka, Japan). Specific primer pairs were designed using Primer3 Input 0.4.0 (http://frodo.wi.mit.edu/primer3/) to amplify fragments between 150 and 200 bp in the non-conserved region. To quantify the expression level of antioxidative enzymes (SOD, CAT and APX), as well as PM H^+^-ATPase and Ca^2+^-ATPase, the rice actin gene (NM 197297) was used as an endogenous control gene. The specific primers for actin were forward 5’-TTATGGTTGGGATGGGACA-3’ and reverse 5’-AGCACGGCTTGAATAGCG-3’. The sequences of the genes *OsCu/Zn SOD1*, *OsCu/Zn SOD2*, *OsAPX1*, *OsAPX2*, *OsACA1*, *OsACA2*, *OsACA3*, *OsACA4*, *OsACA6*, *OsACA7*, *OsACA8*, *OsACA11*, and *OsACA12* were obtained from the Rice Genome Annotation Project (RGAP; http://rice.plantbiology.msu.edu/index.shtml). The gene sequences of *OsCATA*, *OsCATB*, *OsA1*, *OsA2*, *OsA3*, *OsA7*, *OsA8*, and *OsA9* were obtained from the National Center for Biotechnology Information (NCBI; www.ncbi.nlm.nih.gov). The primers used in RT-qPCR are provided in S1 Table. PCR reactions were prepared in 25-μL volumes containing 2 μL of 10-fold diluted synthesized cDNA, 13 μL SYBR Green Realtime PCR Master Mix, 1 μL 10 μM forward primer, 1 μL 10 μM reverse primer, and 8 μL sterile distilled water. Four replications per sample were carried out in parallel, and data analysis was performed as described by Pfaffl [33].

### Statistics

Data were analyzed by one-way analysis of variance with a general linear model using SAS version 9.1 (SAS Institute, Cary, NC, USA). Means were presented with standard errors to indicate variation. Differences between means were determined by *t*-tests (*P*<0.05).

## Results

### Effects of different pH levels on the growth and root morphology of rice

**Fig 1:**
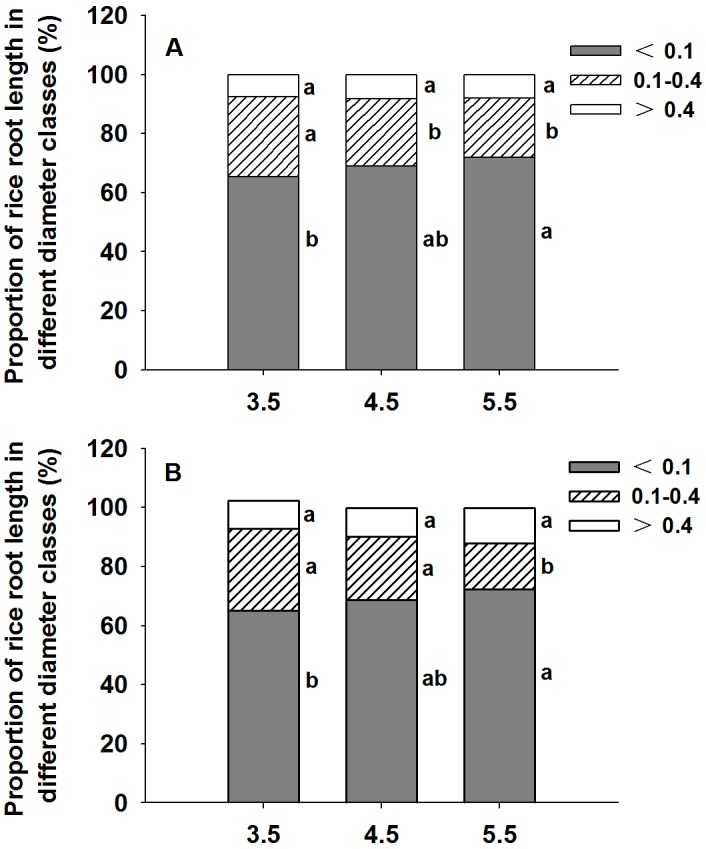
Proportion of rice (*Oryza sativa* L. A, YY12; B, ZZY1) root lengths in different diameter classes at three pH levels. Each value is the mean ± standard error of three replicates. Different letters for each cultivar indicate means that differ significantly (*P* < 0.05) within a given diameter class.

The growth of rice was markedly inhibited by high H^+^ activity (low pH) in the medium (Table 1). Low pH significantly decreased the root dry weight, root length and root surface area of YY12 and ZZY1 compared with the control (pH 5.5; *P* < 0.05). Excess H^+^ significantly reduced the production of fine roots in the two rice varieties (Table 1 and Fig. 1). The proportion of roots in YY12 and ZZY1 with a diameter < 0.1 mm was 10% and 11% less in pH 3.5 medium than in pH 5.5 medium (Fig. 1). The proportion of roots in the diameter class 0.1–0.4 mm was greater in pH 3.5 medium compared with pH 5.5 medium (Fig. 1). No difference was found between pH 4.5 and pH 5.5 in the proportion of roots in each of the three diameter classes.

**Table 1:** Biomass and root morphology of rice (Oryza sativa L.) seedlings grown at different pH levels.

	**pH Treatment**	**Shoot dry weight (g plant^-1^)**	**Root dry weight (g plant^-1^)**	**Root length (cm)**	**Root surface area (cm^-2^)**	**specific root length (m g^-1^)**
YY12	3.5	0.31±0.015b	0.022±0.0012c	717±39c	40±2.35b	331±4.38b
	4.5	0.37±0.027b	0.033±0.003b	1226±83b	69±6.98b	366±10.79a
	5.5	0.47±0.023a	0.050±0.002a	1821±135a	100±11.40a	363±6.40a
ZZY1	3.5	0.49±0.025b	0.034±0.002b	542±81c	33±5.31b	155±5.39c
	4.5	0.57±0.033b	0.044±0.005b	950±115b	57±9.19b	209±21.26b
	5.5	0.69±0.013a	0.065±0.005a	1947±93a	117±7.46a	300±10.12a

Note: Each value is the mean ± standard error of three replicates. Different letters for each cultivar indicate means that differ significantly (P < 0.05).

### Effects of different pH levels on H_2_O_2_ production and lipid peroxidation in roots

**Fig 2:**
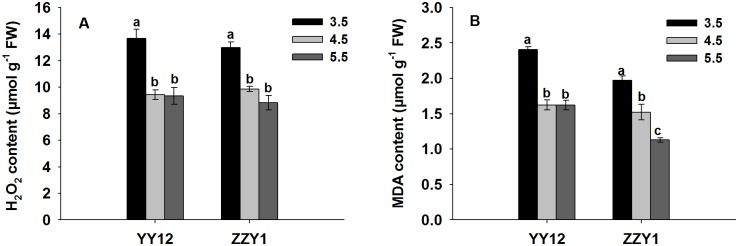
H_2_O_2_ content (A), and MDA content (B) in roots of rice (*Oryza sativa* L.) grown at three pH levels. Each value is the mean ± standard error of three replicates. Different letters for each cultivar indicate means that differ significantly (*P* < 0.05).

Low pH values of the root medium caused an increase in ROS generation in the rice. Compared with pH 5.5, the H_2_O_2_ content of YY12 and ZZY1 roots grown at pH 3.5 was significantly higher (by 45% and 47%, respectively), and increased with increasing H^+^ concentration (Fig. 2). The level of lipid peroxidation in low pH-treated rice plants was measured by the MDA content. Compared with pH 5.5, the low pH significantly increased the MDA content in YY12 and ZZY1 roots by 48% and 74%, respectively.

### Effects of different pH levels on antioxidant enzyme activities and related gene expression levels in rice roots

**Fig 3:**
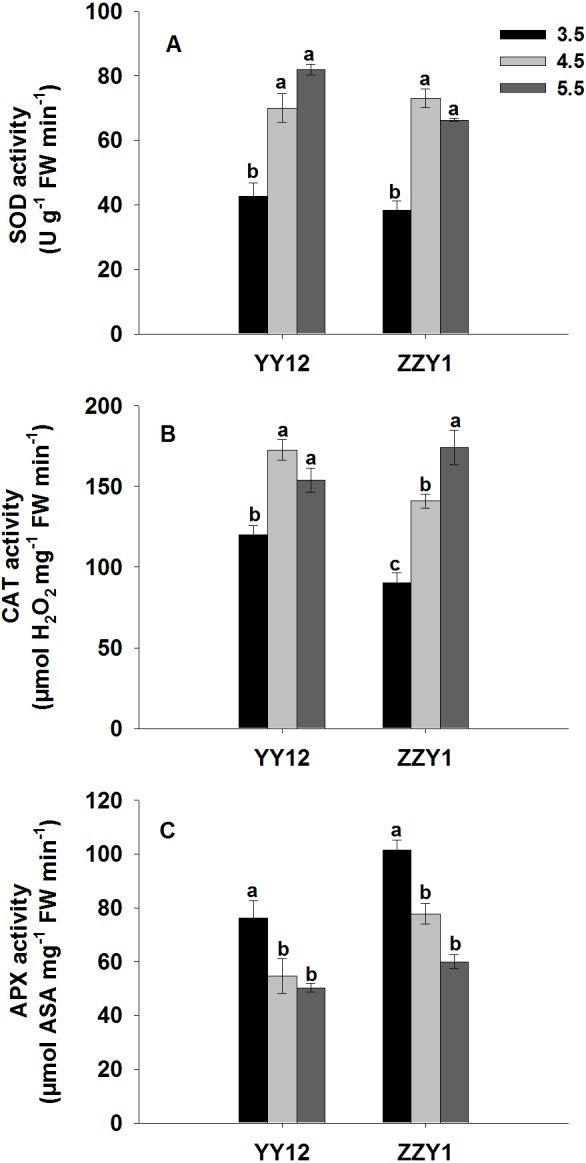
The activities of SOD (A), CAT (B) and APX (C) in roots of rice (*Oryza sativa* L.) grown at three pH levels. Each value is the mean ± standard error of three replicates. Different letters for each cultivar indicate means that differ significantly (*P* < 0.05).

Compared with pH 5.5 medium, the SOD activity of YY12 and ZZY1 in pH 3.5 medium were decreased by 48% and 42%, respectively, and the CAT activity of the rice roots in pH 3.5 medium were decreased by 22% and 48%, respectively. Contrary to the change of SOD and CAT activities, APX activity in YY12 and ZZY1 roots were markedly enhanced by 51% and 69% respectively, on the 14th day of treatment under low pH (Fig. 3).

**Fig 4:**
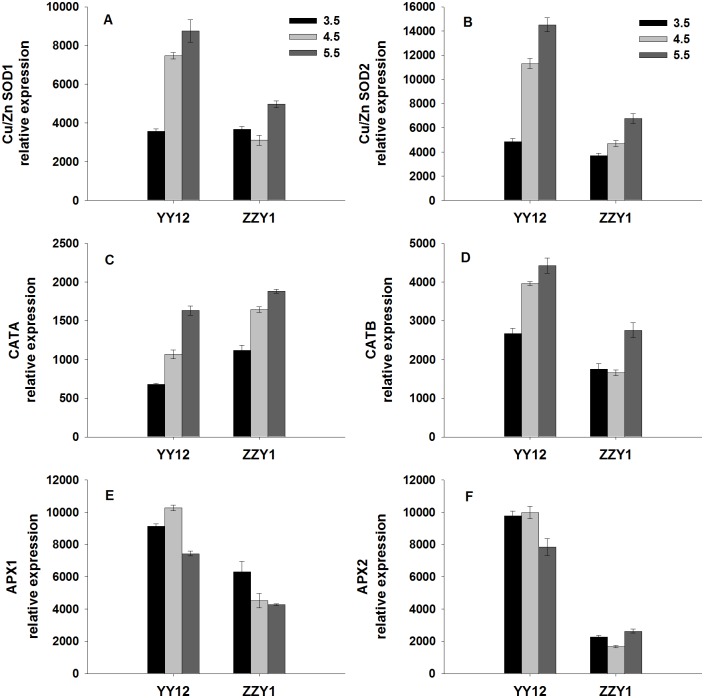
Relative expression ratios (ratio of each gene to actin) of genes encoding antioxidant enzymes (Cu/ZnSOD, CAT, APX) in the roots of rice (*Oryza sativa* L.) grown at three pH levels. Each value is the mean ± standard error of three replicates. Different letters for each cultivar indicate means that differ significantly (*P* < 0.05).

To further clarify the effects of protons on antioxidant enzymes, the mRNA expression levels of *OsCu/Zn SOD*, *OsCAT* and *OsAPX* genes were analyzed with RT-qPCR. The results revealed that the transcript levels of *OsCu/Zn SOD1*, *OsCu/Zn SOD2*, *OsCATA* and *OsCATB* in YY12 and ZZY1 roots decreased under the low pH treatment. However, as shown in Fig. 4, a significant increase was found in the relative expression levels of the *OsAPX1* gene in YY12 and ZZY1 roots exposed to low pH treatment compared with the control. There was no significant change in the relative expression levels of *OsAPX2* in ZZY1 roots exposed to the low pH.

### Effects of different pH on PM ATPase activities and related gene expression levels in roots

**Fig 5:**
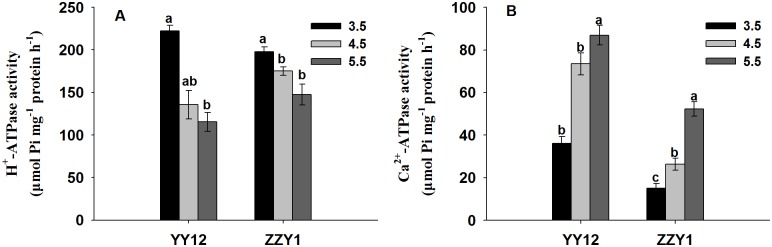
Plasma membrane H^+^-ATPase (A) and Ca^2+^-ATPase (B) activities derived from rice seedling in the roots of rice (*Oryza sativa* L.) grown at three pH levels. Each value is the mean ± standard error of three replicates. Different letters for each cultivar indicate means that differ significantly (*P* < 0.05).

PM H^+^-ATPase and Ca^2+^-ATPase activities in rice roots were measured on the 14th day of treatment and showed different change tendencies. Compared with the control, the PM H^+^-ATPase activities of YY12 and ZZY1 increased significantly (*P* < 0.05) under low pH; however, excess H^+^ significantly inhibited PM Ca^2+^-ATPase activities in the roots of both varieties (Fig. 5).

**Fig 6:**
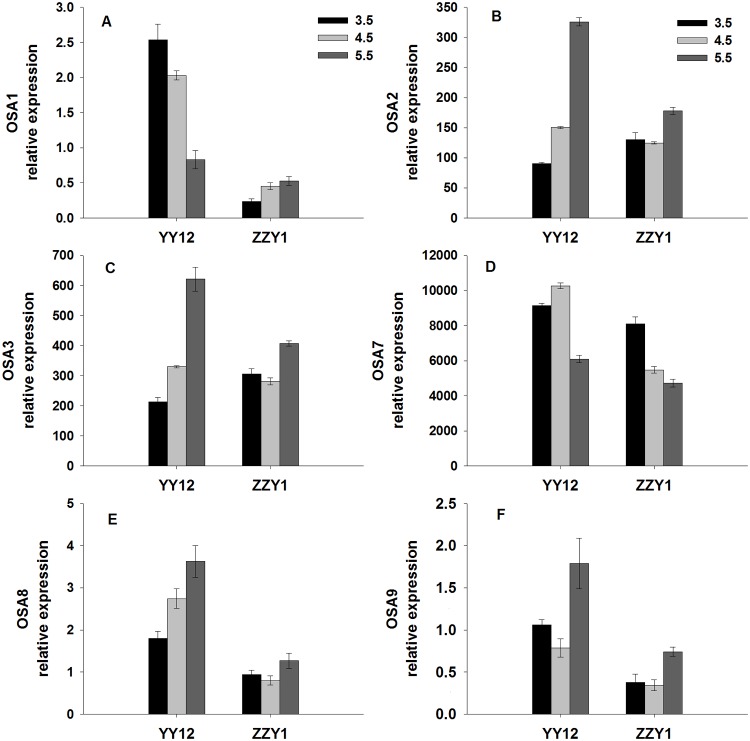
Relative expression ratios (ratio of each gene to actin) of genes encoding plasma membrane H^+^-ATPase in the roots of rice (*Oryza sativa* L.) grown at three pH levels. Each value is the mean ± standard error of three replicates. Different letters for each cultivar indicate means that differ significantly (P < 0.05).

**Fig 7:**
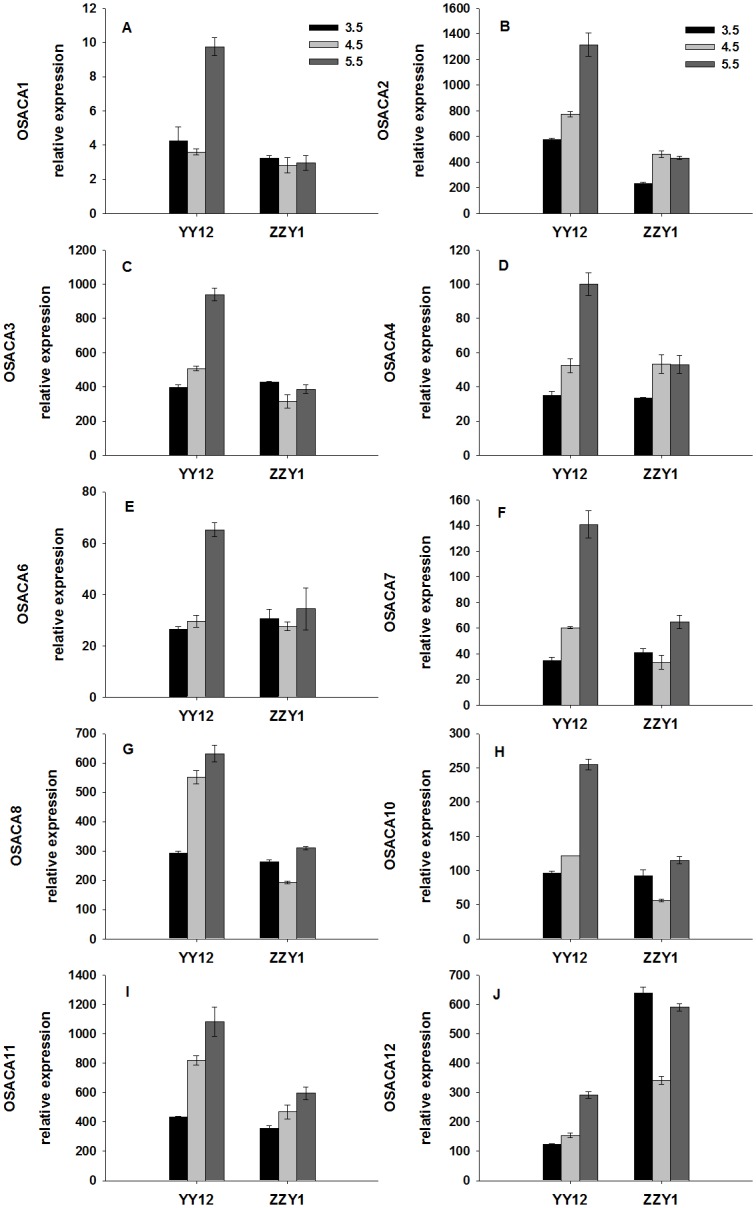
Relative expression ratios (ratio of each gene to actin) of genes encoding plasma membrane Ca^2+^-ATPase in the roots of rice (*Oryza sativa* L.) grown at three pH levels. Each value is the mean ± standard error of three replicates. Different letters for each cultivar indicate means that differ significantly (P < 0.05).

As shown in Fig. 6, the expression levels of six PM H^+^-ATPase genes in rice roots were assayed by RT-qPCR. The expression levels of *OsA2*, *OsA3*, *OsA8* and *OsA9* in YY12 and ZZY1 roots was decreased under low pH compared with the control. The expression of *OsA7* in roots was about 10–20 times higher than the other isoforms. The expression level of *OsA7* in YY12 and ZZY1 under the low pH treatment was markedly higher than in the control. Ten PM Ca^2+^-ATPase genes were detectable, and the expression levels of all 10 (*OSACA1*, *OSACA2*, *OsACA3*, *OsACA4*, *OsACA6*, *OsACA7*, *OsACA8*, *OsACA10 OsACA11* and *OsACA12*) were down-regulated in YY12 roots at pH 3.5 compared with pH 5.5 (Fig. 7). The expression levels of *OSACA2*, *OsACA4*, *OsACA7* and *OsACA11* were down-regulated in ZZY1 roots at pH 3.5 compared with pH 5.5. No significant changes were detected in the relative expression levels of *OSACA1*, *OsACA3*, *OsACA6*, *OsACA8* and *OsACA11* in ZZY1 roots exposed to low pH.

## Discussion

Highly acidic soil pH is one of the major limiting factors in acid soils, and severely inhibits world rice production [5]. In the present study, when treated with a low pH solution, pronounced symptoms were observed in the root system, and the root growth of rice was markedly depressed (Table 1). The root length of YY12 and ZZY1 was reduced and the development of fine roots was suppressed under low pH (Fig. 1). Excess H^+^ not only prohibited metabolic processes, but also affected detrimental oxidative processes in the tissue [12, 34].

It has been reported that abiotic stress often causes symptoms associated with oxidative stress and membrane lipid peroxidation, which can result in the accumulation of ROS and MDA [17, 35, 36]. Here, we found that exposure to low pH significantly increased the H_2_O_2_ content in the roots of YY12 and ZZY1 rice seedlings (Fig. 2). Chen et al. [37] reported that the H_2_O_2_ accumulation in roots could be one reason for leading to the reduction of root elongation. The accumulation of ROS can damage cellular membranes by lipid peroxidation [8, 13, 35]. MDA content is widely used as an indicator for lipid peroxidation [9, 38]. In this study, low pH stress triggered an increase of MDA in the roots of both rice varieties (Fig. 2). These results suggest that lipid peroxidation in rice is an important cue for the inhibition of root elongation and the growth of shoots.

Plants have developed antioxidative systems to minimize the oxidative damage under unfavorable environmental conditions. Here, the SOD and CAT activities in YY12 and ZZY1 roots were obviously down-regulated under low pH. However, the APX activities of YY12 and ZZY1 roots were significantly up-regulated by low pH (Fig. 3). These results imply that APX may play an important role in enhancing plant resistance to low pH by abolishing H_2_O_2_ accumulation. Previous reports have shown that low pH stress also decreases the activities of SOD and CAT in cucumber [8]. Decreased SOD and CAT activities indicate that the ability to scavenge singlet oxygen and H_2_O_2_ in rice is weakened by low pH stress, which may result in free radical-mediated damage, including lipid peroxidation in membranes [39]. In this study, genes encoding antioxidant enzymes exhibited different expression patterns in response to H^+^ stress. The transcript levels of *OsCu/Zn SOD1*, *OsCu/Zn SOD2*, *OsCATA* and *OsCATB* were significantly inhibited in YY12 and ZZY1 roots, whereas the expression of *OsAPX1* was strongly enhanced by H^+^ stress (Fig. 4). It seems likely that the induction of *OsAPX* expression has an important function in removing H_2_O_2_ and minimizing oxidative damage. Rossel et al. [40] reported that an *Arabidopsis thaliana* gain-of-function mutant with constitutively higher *OsAPX2* expression was more drought-tolerant than wild-type plants.

PM H^+^-ATPase plays an important role in membrane potential maintenance during plant responses to various environmental stresses [41]. When roots were exposed to low pH, the inside-negative PM electric potential becomes a driving force for H^+^ uptake and inhibits the growth of the plant. The activity of PM H^+^-ATPase is strongly dependent on pH changes with the optimal pH around 6.6, and is altered at lower cytoplasmic pH [42]. In this study, the H^+^-ATPase activities of YY12 and ZZY1 roots were increased, which implies that induction of H^+^-ATPase activity might play a central role in rice root tolerance to H^+^ stress. This may facilitate the expulsion of excess H^+^, promote cytoplasmic alkalinization and partly restore normal cell activity. Plant PM H^+^-ATPase is encoded by a multigene family [16]. Among the six *OsA* genes examined in the present study, *OsA1*, *OsA2* and *OsA3* belong to subfamily I, *OsA7* belongs to subfamily II, and *OsA8* and *OsA9* belong to subfamilies V and III. The expression of six PM H^+^-ATPase genes in rice roots may help regulate PM H^+^-ATPase activity under different environmental conditions [20]. Four of these genes responded with a decrease in transcription rate when the roots were exposed to low pH. However, the expression of *OsA7* was enhanced and resulted in an increase in enzyme concentration and higher H^+^-pumping activity (Fig. 6). Previous results have indicated that PM H^+^-ATPase is involved in the maintenance of cytosolic pH, especially under long-term acid stress [43, 44]. In this experiment, the expression levels of four PM Ca^2+^-ATPase genes in YY12 and ZZY1 roots were down-regulated when exposed to excess H^+^ (Fig. 7), and low pH inhibited PM Ca^2+^-ATPase activity. Our results suggest that severe membrane lipid damage occurred in the rice roots under low pH stress. The membrane lipid oxidative injury caused by ROS was markedly increased due to the lower activities of anti-oxidative enzymes under H^+^ stress. The lipid damage is one important factor exerting an effect on Ca^2+^-ATPase under stress conditions [45]. As a result of lipid peroxidation induced by excess H^+^, PM Ca^2+^-ATPase proteins were disturbed.

In summary, we found that high H^+^ concentrations significantly suppressed root growth, reduced the development of fine (small diameter) roots and decreased the biomass of rice seedlings. Meanwhile, there were significant increases in lipid peroxidation and the H_2_O_2_ concentration in rice seedlings after low pH treatment for 2 weeks. The gene expression levels of *OsCu/Zn SOD1*, *OsCu/Zn SOD2*, *OsCATA*, *OsCATB* and *OsACA2*, *OsACA4*, *OsACA7*, *OsACA11* in rice roots were significantly down-regulated by strong rhizosphere acidification. Meanwhile, the activities of SOD, CAT and PM Ca^2+^-ATPase were markedly inhibited by higher H^+^ activity in the rice roots. However, increased H^+^ induced higher expression of *OsAPX1* and *OsA7*. Thus, the activation of APX and PM H^+^-ATPase activities in roots may play a key role in scavenging ROS and contribute to the adaptation of rice roots to low pH.

## Supporting Information

S1 Table Candidate reference rice (*Oryza sativa* L.) genes and primers derived from RT-qPCR analysis. (DOC)
