# Supplementary material for: Low pH-Induced Changes of Antioxidant Enzyme and ATPase Activities in the Roots of Rice (Oryza sativa L.) Seedlings
Source: PLoS One. 2015 Feb 26;10(2):e0116971. doi: 10.1371/journal.pone.0116971 (PMC4342341; doi:10.1371/journal.pone.0116971)
Supplement: S1 Table — (DOC) [file pone.0116971.s001.doc]

**Supplementary Table S1. Candidate reference rice (*Oryza sativa*** L.) genes and primers derived from RT-qPCR analysis

| Gene name | GenBank accession number | Primer sequence (5'-3') (forward/reverse) |
| --- | --- | --- |
| OsCu/Zn SOD1 | LOC_Os03g22810.1 | 5'TGTCCAAGAGGGAGATGGTC3' |
|  |  | 5'ATCTTCTGGTGCTCCATGCT3' |
| OsCu/Zn SOD2 | LOC_Os07g46990.1 | 5'ACAGCCAGATCCCCCTTACT3' |
|  |  | 5'TACGAGCGAACATGAACAGC3' |
| OsAPX1 | LOC_Os03g17690.1 | 5'CCAAGGGTTCTGACCACCTA3' |
|  |  | 5'CAAGGTCCCTCAAAACCAGA3' |
| OsAPX2 | LOC_Os07g49400.2 | 5'AAGTGACAAAGCCCTCATGG3' |
|  |  | 5'TCCTCAGCAAATCCCAGTTC3' |
| OsACA1 | LOC_Os01g71240 | 5'TCGTGTTGCACTCTTCGTTC3' |
|  |  | 5'GGAATCTCAGGCTCTTGGTG3' |
| OsACA2 | LOC_Os02g08010 | 5'TCGCCTGTTGTTTCCTCTCT3' |
|  |  | 5'ATGAAGCTCAACACCCCAAG3' |
| OsACA3 | LOC_Os02g08018 | 5'TAGGGCTCATGCTCAAGTCA3' |
|  |  | 5'CACCGTCGTACCATCCTTCT3' |
| OsACA4 | LOC_Os03g10640 | 5'CTCACGACCAACCACATGAC3' |
|  |  | 5'GACTCCCGCTCTTGTTGAAG3' |
| OsACA6 | LOC_Os04g51610 | 5'GACAAGTCCACGTTCAGCAA3' |
|  |  | 5'GCATTTCGTCTTGCAGTCAA3' |
| OsACA7 | LOC_Os05g41580 | 5'TGTCAAGTTTGTTGGGGACA3' |
|  |  | 5'CATCGTCTCCCTTCACCACT3' |
| OsACA8 | LOC_Os08g40530 | 5'TGACGTGCCTCTAAATGCTG3' |
|  |  | 5'AAATGGCCTGGACAAACAAG3' |
| OsACA10 | LOC_Os11g04460 | 5'AGCAGGCTGCACTCATTTTT3' |
|  |  | 5'CCATGATCAAACGATGAACG3' |
| OsACA11 | LOC_Os12g04220 | 5'TTGCAGTCATAGCAGCAACC3' |
|  |  | 5'ATTGGCAAGTGGCCTGTATC3' |
| OsACA12 | LOC_Os12g39660 | 5'TGGAGAAGATCAACGTGCTG3' |
| OsCATA | AK099923 | 5'CGGATAGACAGGAGAGGTTCA3' |
|  |  | 5'AATCTTCACCCCCAACGACT3' |
| OsCATB | AK069446 | 5'GGCAAGATCGTTTTCTCCAG3' |
|  |  | 5'TGGTTTCAGGTTGAGACGTG3' |
| OsA1 | AJ439999 | 5' GTGTTTGGGTTTATGCTGCT 3' |
|  |  | 5' GTATCCACCCAGCACAACTC 3' |
| OsA2 | AJ440000 | 5' GGCTTTGCCAGTTGATGAAT 3' |
|  |  | 5' TGCAAGCGTTTCACAATCTC 3' |
| OsA3 | AF110268 | 5' GCAGATTCTCCACCTTGCTC 3' |
|  |  | 5' GTGCCTTGGAGGATCAAAAA 3' |
| OsA7 | AJ440218 | 5' TTGGTTCCCGGTGACATTAT 3' |
|  |  | 5' CACCAGGGTTCTTGGTCACT 3' |
| OsA8 | AJ440219 | 5'TCGCTCGCTGTTGCCTATCA 3' |
|  |  | 5'TTCCTAACCTCCGGCCTGTC3' |
| OsA9 | AJ440220 | 5'GGTGCGATTGTTGAGGTTAT3' |
|  |  | 5'GTGAGATGGTTGAGGGTGAG3' |
